# Synergistic Fermentation of *Pichia kluyveri* and *Saccharomyces cerevisiae* Integrated with Two-Step Sugar-Supplement for Preparing High-Alcohol Kiwifruit Wine

**DOI:** 10.3390/metabo14060310

**Published:** 2024-05-28

**Authors:** Qiang Wu, Qiaoling Yuan, Xi Wang, Lingying Chen, Senlin Yi, Xiaodan Huang, Jun Wang, Xutong Wang

**Affiliations:** 1 College of Food and Chemical Engineering, Shaoyang University, Shaoyang 422000, China; yqiaolng@163.com (Q.Y.); jennie20040819@163.com (X.W.); c176578007@163.com (L.C.); 17779585628@163.com (S.Y.); hxd18296779538@163.com (X.H.); 15345915026@163.com (J.W.); wangxutong2002@163.com (X.W.); 2 Hunan Key Laboratory of New Technology and Application of Ecological Brewing, Shaoyang 422000, China; 3 Shaoyang Engineering Technology Research Center of Functional Fertilizer, Shaoyang 422002, China

**Keywords:** kiwifruit wine, wild yeast, 26S rDNA D1/D2, synergistic fermentation, *Pichia kluyveri*, two-step sugar supplement

## Abstract

Wild yeast suitable for kiwifruit wine fermentation was isolated and purified, and the fermentation process was optimized to increase the alcohol content of the kiwifruit wine. *Pichia kluyveri* was isolated from kiwifruit pulp by lineation separating, screened by morphological characteristics in Wallerstein Laboratory Nutrient Agar (WL) medium and microscope observation, and further identified by 26S rDNA D1/D2 domain sequence analysis. Taking alcohol content and sensory evaluation as two indexes, the fermentation condition for kiwifruit wine was optimized by single factor and response surface experiment. The optimal fermentation conditions were optimized as follows: the fermentation temperature was at 24 °C, the initial pH was 3.8, the sugar dosage in second step was 8% (*w*/*w*), and the inoculating quantity of *Pichia kluyveri* and *Saccharomyces cerevisiae* was 0.15 g/L at equal proportion. Under these optimal conditions, the maximum estimated alcohol content was 15.6 vol%, and the kiwifruit wine was light green in color with strong kiwifruit aroma and mellow taste.

## 1. Introduction

Wild kiwifruit is the fruit of a dioecious plant mainly distributed in Qinling Mountain, Hunan Western Mountain, Ba Mountain, and Funiu Mountain in China [1]. Fruit wines have gained popularity in recent years due to their unique flavors and aromas. Among these, kiwifruit wine stands out for its distinctive taste and potential health benefits. The production of high-quality kiwifruit wine involves a complex fermentation process that requires the careful control of various factors such as temperature, pH, and sugar supplementation. Compared with artificially cultivated ones, wild kiwifruit not only contains higher vitamin C but is also rich in microorganisms [2]. Lactic acid bacteria isolated from naturally fermented wild kiwifruit wine have been shown to positively impact acid reduction and enhance quality during malic acid-lactic acid fermentation [3]. An exceptional yeast strain was extracted from the peel of wild kiwifruits in Dabie Mountain; although its species remains unidentified, it is suitable for producing kiwifruit wine [4]. *Pichia kudriavzevii* obtained from selenium-rich wild kiwifruits in Sichuan province possesses distinct characteristics such as a rich fruit flavor and strong fermentation capabilities [5]. In our previous study, six wild yeasts were isolated from wild kiwifruits in Great Western Hunan; we also discovered at least six other categories present within the kiwifruits themselves [6]. *Saccharomyces cerevisiae* can be utilized for producing fruit wine with an alcohol content of 7.8 vol% while exhibiting angiotensin I-converting enzyme (ACE) inhibitory activity [7]. Wild kiwifruits are small (30–50 g) and have no advantage in the fresh market for sale purposes [8]. Improving the development and added value of wild kiwifruits is of great significance.

In the process of brewing fruit wine, yeast strains play a crucial role in determining the quality of the final product, encompassing its taste, flavor, and alcohol content. Angel yeast SY is widely utilized commercially for fruit wine production; however, due to the absence of specialized yeasts for kiwifruit fermentation, there is no guarantee regarding the yield and quality of kiwifruit wine. Traditional fermentation methods involve adding activated yeast to fruit juice for fermentation. Nonetheless, the amount of sugar that can be metabolized by yeast is limited, resulting in a generally low alcohol content in fruit wines. Nevertheless, it is important to avoid excessively high initial sugar levels as yeast struggles to survive under such conditions. Therefore, sugar supplementation during fermentation becomes necessary. Two-step fermentation has been extensively employed in chitinase [9] and microbial oil [10] production as it encompasses both bacterial growth stages and induction product production stages which reduce inhibitory effects on bacterial metabolism caused by products or raw materials thereby increasing product yield. With advancements in kiwifruit cultivation technology leading to an oversupply situation in China, effective measures need to be taken to prevent wastage caused by spoilage over time due to excessive supply of fruits. This necessitates developing new brewing technologies along with screening and application of specific strains suitable for kiwifruit fermentation purposes.

*Pichia kluyveri* and *Saccharomyces cerevisiae* are two yeasts commonly used in wine fermentation. *Pichia kluyveri* is known for its ability to produce fruity and floral aromas in wine. It is often used in conjunction with other yeasts to enhance the complexity of the wine’s flavor profile. *Pichia kluyveri* also has the ability to produce higher levels of glycerol, which can enhance the mouthfeel and overall texture of the wine [11]. *Saccharomyces cerevisiae*, on the other hand, is the most common yeast used in winemaking. It is responsible for converting sugars into alcohol and carbon dioxide (CO_2_) during fermentation. *Saccharomyces cerevisiae* also produces a wide range of flavor compounds, including esters, higher alcohols, and sulfur compounds, which contribute to the overall aroma and flavor of the wine [12]. *Pichia kluyveri* can help to enhance the fruity and floral aromas of the wine, while *Saccharomyces cerevisiae* can contribute to the overall fermentation process and flavor profile [13]. Fermentation temperature, pH levels, and sugar control play crucial roles in determining the quality and sensory characteristics of fruit wine. Optimal fermentation temperature and pH levels can lead to a higher alcohol content and desirable sensory attributes, while precise sugar supplementation strategies, can contribute to a more balanced and flavorful wine [14]. Understanding the impact of these factors on the fermentation process is essential for producing high-quality fruit wines with unique sensory profiles.

In this study, *Pichia kluyveri* and *Saccharomyces cerevisiae* worked together synergistically to create a complex and balanced kiwifruit wine. Furthermore, the effects of fermentation temperature, pH levels, and sugar supplementation strategies of the synergistic fermentation of *Pichia kluyveri* and *Saccharomyces cerevisiae* on the quality of kiwifruit wine were investigated followed by optimizing the production of this kiwifruit wine and improving its sensory characteristics. The Greater Xiangxi region serves as one of China’s primary areas for kiwifruit production; hence, this study focuses on utilizing wild kiwifruits from Great Western Hunan as raw material for isolating excellent yeasts suitable for preparing kiwifruit wine through a two-step sugar supplementation combined with synergistic fermentation using *Pichia kluyveri* and *Saccharomyces cerevisiae* approach providing a theoretical foundation for the efficient utilization of wild kiwifruit.

## 2. Material and Methods

### 2.1. Materials

Wild kiwifruits with the reducing sugar content of 6.2–7.0 g/100 g determined using Fehring’s method at the pH of 3.0–3.3, were purchased from Lang Mountain Planting Base (Shaoyang, China). Honey with the reducing sugar content of 30.0–32.1 g/100 g, was produced from Chengbu Nanshan Ranch (Shaoyang, China). Amplification primers including NL-1 (5′-GCATATCAATAAGCGGAG GAAAAG-3′) and NL-4 (5′-GGTCCGTG TTTCAAGACGG-3′) were synthesized by Beijing Liuhe BGI Co., Ltd. (Beijing, China); CTAB, TE, proteinase K, PCR buffer, dNTP and TaqDNA polymerase were purchased from Beijing Baojieluo Biotechnology Co., Ltd. (Beijing, China).

### 2.2. Isolation and Purification of Wild Yeast Strain

In our previous research, three wild yeast strains (JM1, JM11, and JM12) were screened from wild kiwifruit pulp. JM11 was identified as *Saccharomyces cerevisiae*, which can tolerate extreme fermentation environments including high sugar (300 g/L) and high alcohol content (12 vol%) and high temperature (33 °C). Especially, JM1 presented with the strongest acid resistance, surviving at a pH of 2.5 [7]. Hence, JM11 and JM1 was selected for preparing kiwifruit wine in this study. In order to obtain pure JM1, referring to the method of Chai et al. [15] with modification, ten grams of fresh wild kiwifruit pulp was ground in a sterile mortar, and transferred to a jar filled with 90 mL of sterile water. It was placed at 28 °C for 24 h. Two microliters of kiwifruit pulp fluid were applied on YEPD isolation medium and cultured at 28 °C for 3 days. Typical single colony of JM1 was selected to be plated, and streaked on YEPD medium. It was sub-cultured twice to obtain the purified JM1 strain.

### 2.3. Identification of the Purified JM1 Strain

According to the method of Kong et al. [16], the purified JM1 was stained with methylene blue staining, and its morphology was observed under a microscope. Based on the method of Cheng et al. [17], the strain was streaked and inoculated into WL medium. After culturing at 28 °C for 5 days, the colony morphology and color were observed. Referring to the method of Niu et al. [18] with a slight modification, the DNA of this strain was extracted for PCR amplification. After grinding in liquid nitrogen in a mortar, the purified strain was suspended with 600 μL TE, followed by the addition of 250 μL of 10% SDS. It was then mixed by inversion, added with 3 μL of 20 ng/μL proteinase K, and incubated in a water bath at 37 °C for 1 h. After adding with 150 μL of 5 mol/L NaCl and 150 μL of 2% CTAB in turn, it was incubated in a 65 °C water bath for 20 min. After centrifugation at 12,000 rpm for 20 min at room temperature, an equal volume of isopropanol was added to the supernatant, and left at room temperature for 30 min. After centrifuging at 12,000 rpm and 4 °C for 10 min, the precipitate was suspended with 750 μL of 70% alcohol, and then centrifuged at 12,000 rpm and 4 °C for 2 min. 30 μL of precipitate was dissolved overnight at 4 °C. The PCR amplification program was: 95 °C, 30 s, 35 cycles of 55 °C, 30 s, 70 °C, 1 min; 72 °C, 10 min. The PCR products were sent to Beijing Liuhe BGI Co., Ltd. for 26S rDNA D1/D2 sequencing, and sequence homology alignment (BLAST) was performed in the GenBank nucleic acid sequence database.

### 2.4. Two-Step Sugar Supplementation Combined with Synergistic Fermentation Process

#### 2.4.1. Single-Factor Test

Taking the alcohol content and sensory evaluation of kiwifruit wine as two indicators, the single factor test was performed to verify the effects of fermentation temperature, the initial pH of kiwifruit pulp fluid, second-step sugar supplement amount, and yeast inoculation amount on the quality of kiwifruit wine. Referring to the alcohol content determination method of Yu et al. [19], the kiwifruit fermentation supernatant was added with 100 mL of distilled water for distillation to collect 100 mL of distillate which was determined by a density meter for the alcohol content. The sensory evaluation team consisted of 10 people, which were trained before the sensory evaluation and rated the color, the aroma, and the taste of the fruit wine based on the scoring standards as shown in Table 1.

#### 2.4.2. Different Fermentation Temperature 

The wild kiwifruit pulp was added to 0.1% (*w*/*w*) pectinase, and incubated at 35 °C for 4 h. Then, 5% (*w*/*w*) white sugar was added, and the pH was adjusted to 3.8 using sodium citrate. The purified wild yeast JM1 and *Saccharomyces cerevisiae* were mixed in equal proportion, and inoculated at an inoculum amount of 0.15 g/L. Temperature fermentation was set at 4, 20, 24, 28, 32, and 36 °C for 3 days, respectively. After fermentation for 3 days, 4% (*w*/*w*) sterile honey was added into the fermented fluid as the second-step sugar supplementation. It was fermented at 28 °C for 2 days. The alcohol content determination and sensory evaluation of final fermented liquid was conducted.

#### 2.4.3. Different Initial pH

The wild kiwifruit pulp was added to 0.1% (*w*/*w*) pectinase, and incubated at 35 °C for 4 h. Then, 5% (*w*/*w*) white sugar was added, and the pH was adjusted to 3.2, 3.6, 3.8, 4.0, 4.4, or 4.8 using sodium citrate. It was inoculated with both JM1 and *Saccharomyces cerevisiae* with an inoculation amount of 0.15 g/L in equal proportion. It was fermented at 24 °C for 3 days. Then, 4% (*w*/*w*) sterile honey was added in the second-step sugar supplementation. The second-step fermentation was carried out at 28 °C for 2 days, followed by the alcohol content determination and sensory evaluation.

#### 2.4.4. Different Supplement Amount of the Second-Step Sugar Supplement

The wild kiwifruit pulp was added to 0.1% (*w*/*w*) pectinase, and incubated at 35 °C for 4 h. After adding with 5% (*w*/*w*) white sugar, the pH of pulp fluid was adjusted to 3.8 with sodium citrate, and then inoculated with both JM1 and *Saccharomyces cerevisiae* with an inoculation amount of 0.15 g/L. After fermentation at 24 °C for 3 days, sterile honey was added at different supplement amount such as 4, 7, 10, 13, 16, or 19% (*w*/*w*). The second-step fermentation was carried out at 28 °C for 2 days, followed by the alcohol content determination and sensory evaluation.

#### 2.4.5. Different Inoculation Amount of Wild Yeasts

The wild kiwifruit pulp was added with 0.1% (*w*/*w*) pectinase, and incubated at 35 °C for 4 h. After adding with 5% (*w*/*w*) white sugar, the pH of pulp fluid was adjusted to 3.8 with sodium citrate. It was inoculated with both JM1 and *Saccharomyces cerevisiae* with different inoculation amount, including 0.05, 0.10, 0.15, 0.20, 0.25 g/L. Then, the fermentation was performed at 24 °C for 3 days. Sterile honey was added at a supplement amount of 4% (*w*/*w*). It was fermented at 28 °C for 2 days, and the fermentation supernatant was fermented, its alcohol content was measured and sensory evaluation was carried out.

#### 2.4.6. Response Surface Test

According to the Box–Behnken design principle [20], on the basis of above-mentioned single factor experiment, the fermentation conditions were optimized by response surface test. The alcohol content of kiwifruit wine was used as the response value, and the yeast inoculation amount, initial pH, second-step sugar supplement amount, and fermentation temperature were used as influencing factors. Design Expert software was used to analyze the result of response surface test for the optimal synergistic fermentation process of *Pichia kluyveri* and *Saccharomyces cerevisiae* integrated with two-stage sugar-supplement for preparing high-alcohol kiwifruit wine. The experimental design was shown in Table 2.

## 3. Results and Discussion

### 3.1. Identification of JM1 Strain Purified from Wild Kiwifruit

**Figure 1:**
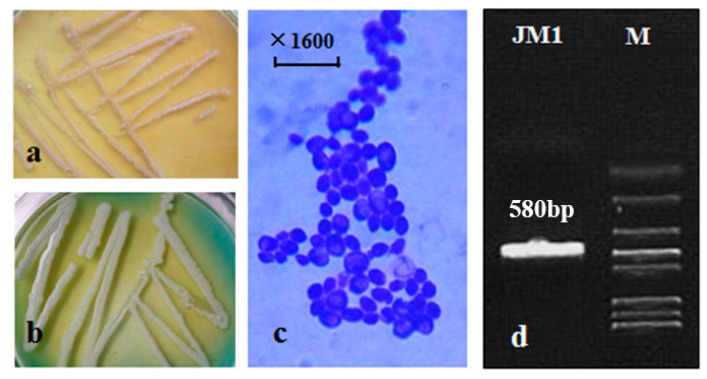
Identification of wild yeast JM1 purified from kiwifruit pulp. (**a**) JM1 colony on YEPD medium; (**b**) JM1 colony on WL medium; (**c**) Cellular morphology; (**d**) Electrophoretogram of PCR product. M: Standard Maker; JM1: PCR product.

**Figure 2:**
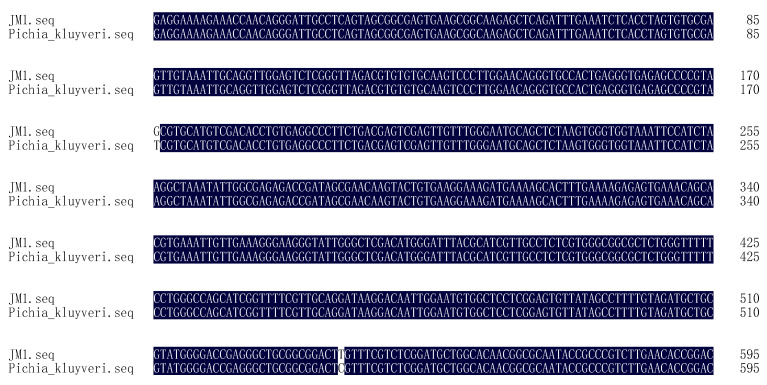
Gene sequence of wild yeast JM1 matched with *Pichia kluyveri*.

The analysis of the JM1 yeast strain isolated from the wild kiwifruit pulp revealed distinct characteristics on the different growth media. On YEPD medium, the colony appeared milky white and small (Figure 1a), while on WL medium, it exhibited a white and smooth appearance with irregular small protrusions on the edges (Figure 1b). Microscopic examination showed that the cell morphology of JM1 was elliptical (Figure 1c). Furthermore, the amplification of the 26S rDNA gene sequence using PCR with primers NL-1 and NL-4 resulted in a product of approximately 580 bp (Figure 1d), which was confirmed by sequencing to be 595 bp in length (Figure 2). Sequence comparison in the GenBank nucleic acid database showed a high homology of 99.6% between this strain and *Pichia kluyveri* (JX103188.1).

The high sequence homology of JM1 with *Pichia kluyveri* further confirmed its classification within this yeast species. The ability of JM1 to produce ester compounds such as isoamyl acetate during fermentation suggests its potential contribution to the aroma and flavor profile of fruit wine [21,22]. The unique colony characteristics observed on different growth media indicated the adaptability of JM1 to varying environmental conditions. The elliptical cell morphology of JM1 is consistent with typical characteristics of *Pichia kluyveri*, further supporting its classification within this yeast species. Understanding the morphology and genetic makeup of JM1 provided valuable insights into its potential fermentation behavior and metabolic activities, particularly in the production of ester compounds that contribute to the sensory attributes of kiwifruit wine. Overall, the findings highlight the importance of yeast selection in wine production and underscore the potential impact of *Pichia kluyveri* (JM1) on the sensory profile of kiwifruit wine. Further research into the fermentation dynamics and metabolic pathways of JM1 can enhance our understanding of its role in flavor development and inform strategies for optimizing kiwifruit wine production. 

### 3.2. Effects of Different Fermentation Conditions on the Quality of Kiwifruit Wine

**Figure 3:**
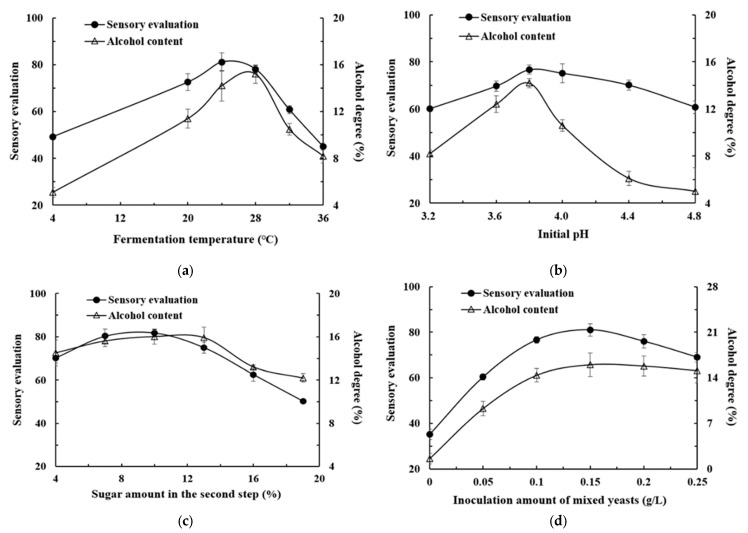
Effects of different fermentation conditions on the quality of kiwifruit wine. (**a**) Fermentation temperature; (**b**) Initial pH; (**c**) Sugar amount in the second step; (**d**) Inoculation amount of mixed yeasts.

The fermentation temperature should be strictly controlled during the brewing of fruit wine [23]. As shown in Figure 3a, when the temperature reached to 28 °C, the alcohol content of kiwifruit wine was 15.2 vol%. However, the temperature was risen to 36 °C, both *Pichia kluyveri* (JM1) and *Saccharomyces cerevisiae* had difficulty surviving, resulting in a low alcohol content in kiwifruit wine. In addition, the alcohol content of kiwifruit wine was only 5.1 vol% at a low temperature of 4 °C. Based on the sensory evaluation results, at a fermentation temperature of 28 °C, the kiwifruit wine was light yellow in color, with a light sour and sweet taste, accompanied by a strong wine aroma and kiwifruit aroma. 

As shown in Figure 3b, when the pH increased to 3.8, the alcohol content reached the highest value of 14.2 vol%, and the sensory score was also the best. Kiwifruit pulp fluid presented relatively acidic, and *Pichia kluyveri* (JM1) and *Saccharomyces cerevisiae* derived from wild kiwifruit were suitable for wild kiwifruit wine brewing. Although the pH was as low as 3.2, the alcohol content of the kiwifruit wine can still reach 8.2 vol%, but the fruit wine was not clear enough and had a strong sour and astringent taste.

During the synergistic fermentation of *Pichia kluyveri* (JM1) and *Saccharomyces cerevisiae* integrated with two-step sugar-supplement for preparing high-alcohol kiwifruit wine in this study, it was only added with a small amount of white sugar in the first step of sugar supplementation. In order to make full use of the sugar contained in kiwifruit by *Pichia kluyveri* (JM1) and *Saccharomyces cerevisiae*, in the second step of sugar supplementation, honey was added to maximize alcohol production. Additionally, the unique flavor of honey made the kiwifruit wine light sour and sweet in taste. However, the sugar content should not be too high, as high osmotic pressure will affect the survival of yeast [24].

As shown in Figure 3c, the alcohol content of kiwifruit wine was increased until the amount of the two-step sugar supplement increased to 10%. After addition with 10% of honey, the alcohol content of kiwifruit wine was 15.4 vol%. When it reached to 13%, the alcohol content was 14.9 vol%. As shown in Figure 3d, the alcohol content of kiwifruit wine was only 1.6 vol% without the inoculation of *Pichia kluyveri* (JM1) and *Saccharomyces cerevisiae*. As the yeast inoculation amount increased, the alcohol content of the fruit wine raised. When the inoculation amount was 0.15 g/L, the alcohol content reached the highest (16.0 vol%). The kiwifruit wine was golden in color, clear and transparent. It had a strong alcohol aroma, moderate sweetness and sourness, and had the best overall sensory experience.

These results highlighted the critical importance of controlling fermentation temperature in kiwifruit wine production in this study. A temperature of 28 °C was identified as optimal for achieving a high alcohol content and desirable sensory attributes in kiwifruit wine. Deviations from this temperature range, such as excessively high (36 °C) or low (4 °C) temperatures, negatively impacted yeast viability, underscoring the need for precise temperature regulation during the brewing process. Furthermore, the pH of the fermentation medium significantly influenced both the alcohol content and sensory characteristics of the kiwifruit wine. A pH of 3.8 was determined to be the most favorable, resulting in the highest alcohol content and sensory score. The acidic nature of kiwifruit pulp fluid supported the fermentation process, indicating the suitability of *Pichia kluyveri* (JM1) and *Saccharomyces cerevisiae* for kiwifruit wine brewing. The synergistic fermentation approach involving *Pichia kluyveri* (JM1) and *Saccharomyces cerevisiae*, combined with a two-step sugar-supplement strategy incorporating honey, demonstrated promising results in enhancing alcohol content and flavor complexity in kiwifruit wine. The careful management of sugar levels, particularly with the addition of honey, contributed to a more balanced and flavorful wine. 

These findings provide valuable insights for optimizing the fermentation process and improving the sensory characteristics of kiwifruit wine, emphasizing the importance of temperature, pH, and sugar control in achieving desired fermentation outcomes. Further research could lead to enhanced strategies for producing high-quality kiwifruit wines with unique sensory profiles.

### 3.3. Optimization of Synergistic Fermentation process of Pichia kluyveri and Saccharomyces cerevisiae

Response surface test was performed to conduct regression analysis on each factor, including fermentation temperature (A), initial pH (B), and second-step sugar supplement amount (C) and wild yeast inoculum (D). The results are shown in Table 3, and the regression equation prediction model was as follows:

Y = 13.68 + 0.62A − 1.83B + 0.67C + 0.35D−0.65AB + 0.15AC + 0.82AD + 0.40BC − 0.075BD + 0.75CD − 0.93A^2^ −2.58B^2^ −0.49 C^2^ −1.02D^2^.

Variance analysis on the regression equation was performed with the alcohol content *Y* as the response value (Table 4). The *F* value of the model was 9.29 (*p* < 0.0001), indicating that the quadratic model used in this test was extremely statistically significant. The lack of fit value was used to indicate the degree of fit between the used model and the experiment [25]. The lack of fit value *p* was 0.8772, higher than 0.05. It indicated that the fit was good, and there is no lack of fit factor. The linear coefficient R^2^ was 0.9028, suggesting that the linear relationship between the dependent variable and the independent variable was significant. The corrected regression coefficient R_Adj_ was 0.8056. It showed that the regression equation had a high degree of fit, and can effectively analyze and predict the effect of alcohol content changes in kiwifruit wine under these process conditions. In addition, the effects of various factors on the alcohol content of kiwifruit wine were ranked as follows: initial pH > sugar amount in the second step > fermentation temperature > inoculation amount.

**Figure 4:**
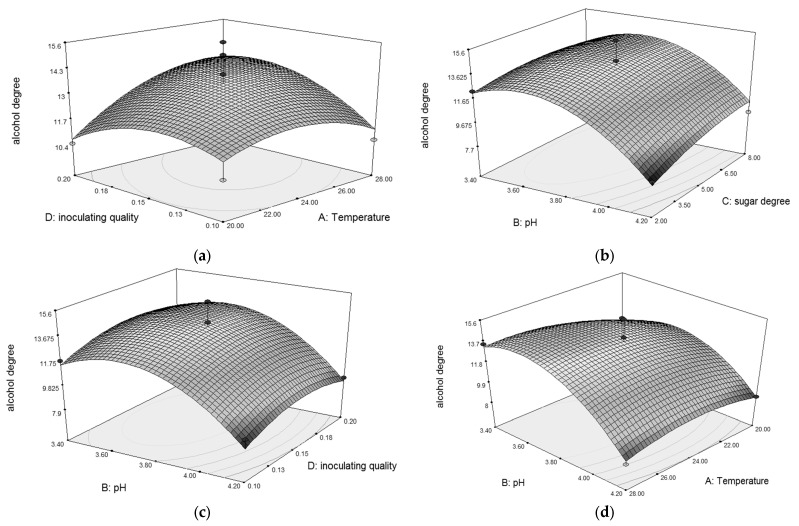
Response surface of the interaction between two factors on alcohol content. (**a**) Inoculation amount of mixed yeasts and fermentation temperature; (**b**) Initial pH and sugar dosage in second step; (**c**) Initial pH and inoculation amount of mixed yeasts; (**d**) Initial pH and fermentation temperature.

Response surface diagram can reflect the strength of the interaction of each factor, and intuitively present the impact of the interaction of two factors on the response value [26]. As shown in Figure 4, the interactions between the inoculum amount and fermentation temperature, and the inoculum amount and the amount of sugar supplement in the second step were relatively obvious. There was no obvious interaction between the initial pH and fermentation temperature, or between the initial pH and the amount of sugar supplement in the second step. The best predicted conditions were obtained as follows: the fermentation temperature was 24.4 °C; the pH was 3.7; the sugar supplement amount in the second step was 8.4% (*w*/*w*); the inoculum amount was 0.13 g/L. Under these optimal conditions, the predicted alcohol content was 15.0 vol%. Considering the actual situation, the fermentation conditions were revised as follows: the fermentation temperature was 24 °C, the pH was 3.8, the sugar supplement amount in the second step was 8.0% (*w*/*w*), and the inoculum amount was 0.15 g/L. The actual alcohol content measured when brewing kiwifruit wine under these conditions was 15.6 vol%, closed to the predicted value (*p* > 0.05). The final reducing sugar content of the kiwifruit wine was determined with 4.25 g/100 g, which was lower than the reducing sugar content of wild kiwi fruit.

Pure fermentation process using commercial yeast or wild yeast is widely applied to brew kiwifruit wine [27,28,29,30,31,32,33,34]. The key fermentation conditions of the isolated yeast from Shanxi kiwifruit peel were optimized as follows: the inoculation amount was 8%; the sugar content was 240 g/kg, resulting in an alcohol content of was 14.14 vol% [27]. The commercial Angel active dry yeast was used to brew kiwifruit wine with an inoculation amount of 1.4%, and the added sugar amount of 20.7%. The alcohol content of the final kiwifruit wine was 10.4 vol% [27]. The red heart kiwifruit wine brewing conditions were as follows: an alcohol content of 12.54 vol%; the inoculum volume was 0.4%; the initial sugar content was 250 g/L [29]. In this study, wild *Pichia kluyveri* (JM1) and *Saccharomyces cerevisiae* were used as a mixed starter culture, accompanied by two-step sugar supplementation. The following advantages of low inoculation amount, low sugar addition, short fermentation cycle, high alcohol content, and good product sensory quality were expected in addition to a greatly reduced manufacturing cost. Furthermore, studying the potential health benefits of kiwifruit wine, such as its antioxidant properties or nutritional content, could be of interest for consumers seeking functional beverages. Understanding the bioactive compounds present in kiwifruit wine and their potential health-promoting effects could add value to the product and increase its market appeal.

## 4. Conclusions

The synergistic fermentation of wild purified *Pichia kluyveri* (JM1) and *Saccharomyces cerevisiae* from kiwifruit pulp have proven to be pivotal in the brewing of high-quality kiwifruit wine. Through single-factor tests and response surface experimental design, we identified the optimal fermentation conditions for kiwifruit wine production. The key parameters included a fermentation temperature of 24 °C, initial pH of 3.8, a sugar amount in the second of 8.0% (*w*/*w*), and an inoculum amount of 0.15 g/L for both yeast strains (*Pichia kluyveri* and *Saccharomyces cerevisiae*). Under these optimized conditions, the kiwifruit wine produced exhibited a remarkable alcohol content of 15.6 vol% and a reducing sugar content of 4.25 g/100 g. The wine was characterized by its clear, golden color, rich aroma, and the distinctive fragrance of kiwifruit, indicating a successful fermentation process that enhanced both the quantitative and qualitative aspects of the final product. The findings from this study lay the foundation for future research in the field of kiwifruit wine production, offering insights into optimizing fermentation parameters and enhancing the sensory attributes of the final product. By continuing to explore innovative approaches and ingredients, there is great potential for developing high-quality kiwifruit wines that cater to diverse consumer preferences and contribute to the growth of the fruit wine industry.

## Figures and Tables

**Table 1:** Sensory evaluation criteria of kiwifruit wine.

Project	Sensory Description	Score
Color	Golden color, transparent, and clear	20–30
	Light yellow color, not bright enough	10–19
	Yellowish brown, not clear enough	5–9
	Grayish brown, not clear	0–4
Fragrant	Strong alcohol scent, fragrant kiwifruit aroma	20–30
	Lighter alcohol scent, slight kiwifruit aroma	10–19
	Very light alcohol scent, slight kiwifruit aroma	5–9
	No obvious alcohol scent	0–4
Taste	Rich wine astringency, light sour and sweet taste	30–40
	Slightly lighter alcohol taste, balanced acidity and wine taste	20–29
	Very light alcohol taste, obvious sourness	10–19
	Strong sour taste, no alcoholic taste	0–9
Total score	--	100

**Table 2:** Sensory evaluation criteria of kiwifruit wine.

Project	Sensory Description	Score
Color	Golden color, transparent, and clear	20–30
	Light yellow color, not bright enough	10–19
	Yellowish brown, not clear enough	5–9
	Grayish brown, not clear	0–4
Fragrant	Strong alcohol scent, fragrant kiwifruit aroma	20–30
	Lighter alcohol scent, slight kiwifruit aroma	10–19
	Very light alcohol scent, slight kiwifruit aroma	5–9
	No obvious alcohol scent	0–4
Taste	Rich wine astringency, light sour and sweet taste	30–40
	Slightly lighter alcohol taste, balanced acidity and wine taste	20–29
	Very light alcohol taste, obvious sourness	10–19
	Strong sour taste, no alcoholic taste	0–9
Total score	--	100

**Table 3:** The response surface experiment result based on Box–Behnken design.

No.	A	B	C	D	Y/vol%	No.	A	B	C	D	Y/vol%
1	20	3.4	8	0.15	11.3	16	24	3.8	8	0.15	13.0
2	24	3.4	8	0.10	11.8	17	20	3.8	6	0.15	11.1
3	28	3.8	10	0.15	14.3	18	20	3.8	8	0.10	10.7
4	20	3.8	8	0.20	10.4	19	24	4.2	10	0.15	8.9
5	28	4.2	8	0.15	8.0	20	24	3.4	10	0.15	11.6
6	24	3.4	6	0.15	12.3	21	24	3.8	10	0.10	12.3
7	24	4.2	8	0.20	8.8	22	24	3.8	8	0.15	14.0
8	24	3.8	8	0.15	15.6	23	24	3.8	8	0.15	12.8
9	24	3.4	8	0.20	12.2	24	24	3.8	8	0.15	13.0
10	24	4.2	8	0.10	8.7	25	28	3.4	8	0.15	13.5
11	24	3.8	10	0.20	14.3	26	20	3.8	10	0.15	12.7
12	20	4.2	8	0.15	8.4	27	24	4.2	6	0.15	8.0
13	28	3.8	8	0.20	13.6	28	24	3.8	6	0.20	10.8
14	24	3.8	6	0.10	11.8	29	28	3.8	6	0.15	12.1
15	28	3.8	8	0.10	10.6						

**Table 4:** Regression simulation and variance analysis of response surface experiment.

Source of Variation	Sum of Square	Degrees of Freedom	Mean Square	*F* Value	*p* Value	Significance
Model	104.83	14	7.49	9.29	<0.0001	
Improper	5.80	10	0.58	0.42	0.8772	
error	5.49	4	1.37			
sum	116.12	28				
R^2^ = 0.9028						
R_Adj_ = 0.8056						
A	4.69	1	4.69	5.81	0.0302	*
B	39.97	1	39.97	49.57	<0.0001	***
C	5.33	1	5.33	6.62	0.0222	*
D	1.47	1	1.47	1.82	0.1983	-
AB	1.69	1	1.69	2.10	0.1697	-
AC	0.09	1	0.09	0.11	0.7432	-
AD	2.72	1	2.72	3.38	0.0874	-
BC	0.64	1	0.64	0.79	0.3880	-
BD	0.02	1	0.02	0.03	0.8697	-
CD	2.25	1	2.25	2.79	0.1170	-
A^2^	5.58	1	5.58	6.92	0.0198	*
B^2^	43.09	1	43.09	53.45	<0.0001	***
C^2^	1.56	1	1.56	1.93	0.1863	-
D^2^	6.68	1	6.68	8.29	0.0121	*

Note: *p* value between 0.01 and 0.05 is shown with one asterisk (*) and *p* value less than 0.001 is shown with three asterisks (***).

## Data Availability

The data presented in this study are available upon request from the corresponding author. The data are not publicly available due to institutional policy regarding data protection.
